# Chemical Composition Analysis of Sea Buckthorn (*Hippophae*) in Georgia and Development of Innovative Valorization Technologies

**DOI:** 10.1002/fsn3.70507

**Published:** 2025-07-14

**Authors:** Lana Datuashvili, Maia Vanidze, Indira Japaridze, Nona Surmanidze, Inga Kartsivadze, Ruslan Davitadze, Aleko Kalandia

**Affiliations:** ^1^ Department of Chemistry, Faculty of Natural Sciences and Health Care Batumi Shota Rustaveli State University (BSU) Batumi Georgia

**Keywords:** green extraction, natural products, Sea buckthorn (*Hippophae*)

## Abstract

Sea buckthorn ( *Hippophae rhamnoides* L.), a wild berry abundant in Georgia, is a rich source of bioactive compounds, including antioxidants, fatty acids, and phenolic compounds, with significant potential for applications in food, nutraceutical, and cosmetic industries. Despite its traditional uses, the physicochemical properties and valorization potential of Georgian sea buckthorn remain underexplored. This study aimed to analyze the chemical composition of sea buckthorn berries from three distinct regions of Georgia (Adjara, Samtskhe‐Javakheti, and Imereti) and develop innovative, environmentally friendly extraction technologies to maximize the recovery of valuable compounds. Using advanced techniques such as ultrasound‐assisted extraction (UAE) and supercritical water extraction (SWE), alongside traditional methods like maceration and Soxhlet extraction, the study identified and quantified 18 bioactive compounds, including organic acids (malic and citric acid), phenolic acids (quinic acid), and flavonoids (e.g., gallocatechin, catechin, and isorhamnetin derivatives). The lipid fraction, predominantly composed of C18 fatty acids (57.4%–76.9%), was rich in linoleic acid (29.4%–50.0%), with extraction yields varying by method. Total phenolic content ranged from 19.90 to 34.94 mg/g dry weight, with higher levels in Samtskhe‐Javakheti and Imereti samples. The highest carotenoid content (3.363 mg/g dry weight) was observed in Samtskhe‐Javakheti berries. UAE with 75% ethanol optimized carotenoid (1.684 mg/g) and phenolic (8.213 mg/g) recovery, while SWE achieved 86% lipid yield and high phenolic content (92.25 mg/g) in just 7 min. Carotenoid‐enriched sunflower oil exhibited 3.5‐fold higher antioxidant activity than control oil, demonstrating enhanced oxidative stability. The study highlights the efficacy of green extraction methods in valorizing sea buckthorn by‐products, offering sustainable solutions for producing high‐value nutraceuticals and functional foods. These findings underscore the potential of Georgian sea buckthorn as a source of natural antioxidants and lipids, with applications in health‐promoting products.

## Graphical abstract

Sea buckthorn, widely found as a wild berry in Georgia, is a rich source of natural antioxidants, traditionally used for preparing food additives like sea buckthorn oil, carotenoids, tocopherols, and phospholipids. Despite its longstanding use, this berry's physicochemical properties and potential applications remain underexplored. This study investigates the biologically active compounds in sea buckthorn berries harvested from different climatic and soil regions across Georgia.

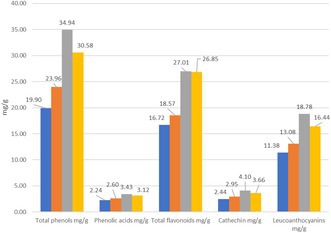

## 1 Introduction

Sea buckthorn ( *Hippophae rhamnoides* L.) is an ancient plant, a deciduous tree or shrub belonging to the Elaeagnaceae family (Li and Schroeder 1996; Dubey et al. 2024; Ilhan et al. 2021). Its wide distribution spans temperate, cold‐temperate, and subalpine regions across Europe and Asia (Mei et al. 2023; Sea Buckthorn Market 2025; Janceva et al. 2022), and it is also found in North America (Canada) (Ilhan et al. 2021). While predominantly found in the wild, sea buckthorn has been actively cultivated in recent centuries (Ilhan et al. 2021).

Almost all parts of the sea buckthorn plant—fruits, leaves, stems, flowers, and seeds—are rich in biomass containing a diverse array of biologically active compounds, which accounts for their wide‐ranging applications in the food, cosmetic, and pharmaceutical industries (Netreba et al. 2024; Chen et al. 2023). The specific utilization of different plant parts is determined by the industrial context. For instance, the fruits are primarily valued for their high content of vitamins, antioxidants, and oils, rendering them ideal for food products and nutritional supplements, while leaves are frequently used to extract compounds for medicinal and cosmetic applications due to their rich profile of phenolic compounds (Chaachouay and Zidane 2024; Olas 2016). Sea buckthorn seeds are particularly important as a source of oil rich in essential fatty acids (linoleic (18∶2ω‐6) and α‐linolenic (18∶3ω‐3) acids) (Fatima et al. 2012). Notably, sea buckthorn seed oil is characterized by its high nutritional value and potential therapeutic applications, attributed to its composition of essential fatty acids and other bioactive components (phenolic acids, flavonoids, and tannins) (Danielski and Shahidi 2024).

The fruit alone contains up to 200 active compounds (Máté et al. 2022; Lee et al. 2021; Criste et al. 2020), including proteins, fiber, antioxidants, vitamins, minerals, phenolic compounds, essential oils, organic acids, and saponins (Dubey et al. 2024; Ilhan et al. 2021). Among the phenolic compounds, significant amounts of flavonols, flavones, phenolic acids, leucoanthocyanidins, and hydrolyzable tannins accumulate, which are the main contributors to the antioxidant activity of sea buckthorn berries and leaves (Li and Schroeder 1996; Mei et al. 2023; Laskowska et al. 2022). Sea buckthorn fruit extracts also exhibit anti‐inflammatory and immunomodulatory properties (Lee et al. 2021; Jaśniewska and Diowksz 2021). These diverse phytochemicals, including flavonoids, organic acids, and phenolic compounds, contribute significantly to its applications in food, health, and cosmetics (Rodríguez‐Negrete et al. 2024; Kumari and Bhargava 2021).

The demand for sea buckthorn products in the global market is rapidly growing, driven by increasing consumer interest in natural and healthy products (Sea Buckthorn Market 2025; El‐Sohaimy et al. 2022; Luntraru et al. 2022; Grabacka et al. 2024). This surge in demand has led to extensive research on the chemical composition of sea buckthorn fruits from various geographical areas and their potential applications (Nybom et al. 2023; Kallio et al. 2002; Ilhan et al. 2021; Korekar et al. 2013; Chen et al. 2013; Ji et al. 2020; Martiniakova et al. 2024; He et al. 2023).

The extraction of these valuable bioactive compounds is a critical step in utilizing sea buckthorn. Traditional extraction methods, such as maceration and Soxhlet extraction, have been employed for many years to study the biologically active compounds in plants. However, modern techniques, including ultrasound‐assisted extraction (UAE) and supercritical fluid extraction (SFE), are increasingly being used due to their higher efficiency, selectivity, and environmental friendliness (Dulyanska et al. 2022; Jia et al. 2018; Paşayeva et al. 2025). The use of effective methods for the extraction of phytochemical compounds ensures the optimization of resources, the maximum extraction of compounds, and the reduction of waste generation. It increases the quality and purity of the resulting extract (Bhardwaj et al. 2021). It also contributes to the economic sustainability and competitiveness of industries by reducing production costs and improving product quality (Jan et al. 2021).

These advancements play a crucial role in connecting fundamental research on plant chemistry to the development of new applications and products. However, studies on “Georgian sea buckthorn” are limited. Sea buckthorn has grown wild in Georgia since ancient times, and its healing properties have been widely recognized in folk medicine. In recent years, interest in sea buckthorn as a promising crop has increased.

The aim of our research was to study the chemical composition of wild sea buckthorn fruits in Georgia, obtain various functional products, and develop innovative valorization technologies.

## 2 Materials and Methods

### 2.1 Plant Material

Wild sea buckthorn was collected in three different regions of Georgia: Samtskhe‐Javakheti (Akhaltsikhe—41°38′20.0″N, 42°59′10.0″E), Adjara (Erge—41°33′41.0″N, 41°41′48.0″E, Tkhilnari—41°33′49.0″N, 41°39′11.0″E) and Imereti (Terjola—42°11′00.0″N, 42°58′38.0″E). Some of the collected samples were stored in a freezer (−25°C) for further studies. Some of them were subjected to direct processing. The samples were also lyophilized and then ground into particles no larger than 250 μm. The processed samples were then stored in a refrigerator at 4°C until use.

### 2.2 Chemicals

The following solvents have been used for analysis: Folin‐Ciocalteau's phenol reagent (Sigma‐Aldrich, 102,664,886, BCCH3316), 2,2‐diphenyl‐1‐picrylhydrazyl (DPPH) (Sigma‐Aldrich), vanillin (Sigma‐Aldrich, V1104‐100G), chlorogenic acid (Sigma‐Aldrich, Lot#SLBJ3632V), caffeic acid (ROTH, Art. Nr.5869.3), (+) catechin hydrate Biosynth, FC30661), β carotene (Sigma‐Aldrich, С4582), n‐hexane (Merck, 1.04391.2500, Germany, CAS‐No: 110–5403), acetic acid (Merck, 1.00063.2511, Germany, CAS‐No: 64‐19‐17), methanol (Roth, Germany, Art.‐Nr. 8388.6), P‐anisidine (Germany, Aldrich, A88255‐100G, Lot # BCCB8823),2,2,4‐trimethylpentanene (Acros organics, Code: 265440010, Lot: A0350543), and potassium hydroxide (Fisher chemical, Cod: P/5600/53, Lot: 1225333). All solvents/chemicals used were analytical or HPLC grade.

### 2.3 The Preparation of Extracts

Various extraction methods were used to isolate substances from the samples: classical maceration with alcohol solutions, Soxhlet extraction for separating lipids, as well as modern, environmentally friendly methods such as ultrasonic and supercritical water extraction. Ultrasound‐assisted extraction (UAE) was performed using an ultrasonic probe processor (Hielscher UP400St, 400 W, 24 kHz). The processor was equipped with an 18 mm sonoprobe and operated in pulsed mode to deliver ultrasonic energy to the solution during extraction. Commercial sunflower oil was also used as an alternative solvent for carotenoids in ultrasonic probe extraction (Surmanidze et al. 2024; Gęgotek et al. 2018; Tan et al. 2022).

### 2.4 UPLC‐MS/MS Analysis

Qualitative analysis of bioactive compounds was done by using ultra‐high‐performance (pressure) liquid chromatography (UHPLC), PDA, and MS methods. Analysis of phenolic compounds by column BEN C18, 1.7 μm, Solvent 1%–0.2% F.A, solvent 2‐ACN, (gradient), Flow 0.3 mL min‐1, column tem. 30°C, MS scan 100–1200 da, Probe 500°C, negative (ESI‐MS)‐, Spray voltage at 0.8 kV, capillary 1.5 kV, CV 5–40 (Surmanidze et al. 2024);

### 2.5 The Determination of Total Carotenoid Content

Total carotenoid content by spectral method. Optical density of the extract treated with a hexane: acetone mixture (4:6) was determined at 480 nm. The results were recalculated using the β‐carotene calibration curve converted to mg/g dry weight (*y* = 0.0916*x* + 0.0042, *R* ^2^ = 0.9993) (Lazzarini et al. 2022; Lasano et al. 2019; Hagos et al. 2022; Visan et al. 2023).

### 2.6 The Determination of Total Phenolic Content

According to the Folin–Ciocalteu method, 1 mL of Folin–Ciocalteu reagent was added to 1 mL of the extract; after 3 min, 1 mL of 10% Na_2_CO_3_ was added, and the volume was brought to 10 mL with water. After 90 min, the optical density was determined at 750 nm. The results were expressed as chlorogenic acid—mg/g sample (*y* = 2.3923*x* + 0.0624, *R* ^2^ = 0.9942) (Kupina et al. 2018; Abashidze et al. 2024; Paşayeva et al. 2022).

### 2.7 Phenolic Acid Content

Spectral method. 250 μL of 0.1% ethanol hydrochloric acid solution and 4.55 mL of 2% hydrochloric acid solution were added to 250 μL of the extract. After vigorously stirring the mixture, we waited 15 min and measured at 320 nm. To convert the absorption value to concentration, the standard calibration curve of caffeic acid (*y* = 4.1338*x* + 0.0393, *R* ^2^ = 0.9986) (Abashidze et al. 2024).

### 2.8 The Determination of Total Flavonoid Content (TFC)

By the AlCl_3_ spectral method. 1 mL of the total extract volume was transferred into a 10 mL test tube, 5 mL of H_2_O, and 0.3 mL of 5% NaNO_2_ were added, and incubated for 5 min. Then, 0.3 mL of 10% AlCl_3_ was added and incubated for 6 min. After this time, 2 mL of 1 N NaOH was added, and the optical density of the solutions was determined at 510 nm. The data obtained as a result of the determination were recalculated into quercetin in mg/g of sample (*y* = 1.1543*x*—0.0023, *R* ^2^ = 0.9992) (Paşayeva et al. 2022; Uranishi et al. 2024; Sultana et al. 2024).

### 2.9 Quantitative Determination of Catechins (TCC)

Add 3 mL of vanillin reagent to 1 mL of the total volume of the extract and determine the optical density of the red‐colored sample at 500 nm after 3 min. 1 mL of the corresponding extractant and 3 mL of vanillin reagent are used as a control. The data obtained as a result of the determination are recalculated onto the calibration curve of (+) catechin (*y* = 33.615*x* − 0.0364, *R* ^2^ = 0.9952) (Dulyanska et al. 2022; Jia et al. 2018; De la Luz Cádiz‐Gurrea et al. 2014).

### 2.10 Quantitative Determination of Leucoanthocyanins (TLC)

8 mL of leucoanthocyanidin reagent is added to 1 mL of the total volume of the extract. A parallel sample is prepared for each sample, which is not heated. The second sample is heated for 40 min. After this time, the samples are determined at 550 nm. The unheated sample serves as a control for the heated sample. The resulting data are converted to mg/g dry weight according to the cyanidin‐3‐O‐glucoside calibration curve (Mannino et al. 2021).

### 2.11 DPPH Radical Scavenging Assay—DPPH Free Radical‐Scavenging Activity Using the Method

DPPH is a stable radical of 2,2‐diphenyl‐1‐picrylhydrazyl, the antioxidant activity of which is determined by 50% inhibition of the sample taken for analysis. The method is based on the determination of the color intensity (517 nm) after 15‐min exposure of the mixture of extract (1 mL) and radical (3 mL). Considering the difference in optical density, sample mass, and extract volume, antioxidant activity was defined as 50% inhibition of DPPH radical per mg of sample (Paşayeva et al. 2022; Gulcin and Alwasel 2023; Wu et al. 2024; Sirivibulkovit et al. 2018).

### 2.12 Lipid Compounds of Sea Buckthorn Fruits

The composition of carboxylic acids was studied using a gas chromatograph (TRACE 1310 Gas Chromatograph—Thermo Scientific). Chromatography was performed on an Agilent J&W CP‐Sil 88 for FAME GC Column/capillary column CP7489 with a length of 100 m, a diameter of 0.25 mm, and a particle size of the stationary phase of 0.20 μm (Surmanidze et al. 2024).

### 2.13 Statistical Analysis

Standard error was calculated for each data point using Excel. Confidence interval *p* ≤ 0.05 (Surmanidze et al. 2024).

## 3 Results

### 3.1 The Determination of Total Phenolic Content (TPC)

**FIGURE 1:**
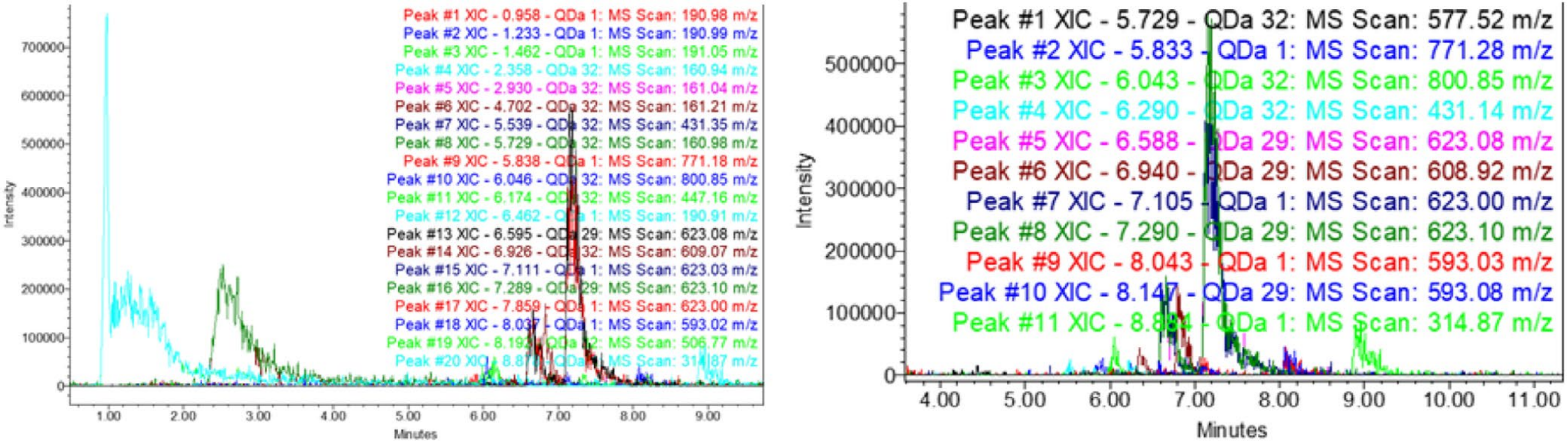
UPLC‐PDA‐MS chromatogram of sea buckthorn fruit and preparations, M/Z (M‐H).

The phenolic composition of sea buckthorn fruits collected from different regions of Georgia was analyzed using an ACQUITY Ultra Performance Liquid Chromatography system (Waters) equipped with mass spectrometry (MS) and a photodiode array (PDA) detector. The compounds were tested for negative ionization at 278 and 350 nm for phenolic acids and flavonoids (Figure 1). Identification was carried out based on standard samples, as well as MS fragmentation, UV spectrum, the METLIN compound mass database, and literature data. [39]. The results are presented in Table 1 and shown in Figure 1.

**TABLE 1:** Biologically active compounds of sea buckthorn by UPLC‐PDA‐MS method.

№	Substance	m/z [M – H}–	Retention times	Berries	The preparation of the pulp	The preparation of the press (skin/seed)
**Organic acid**						
1	Malic acid C_4_H_6_O_6_	133	0.767	+	+	+
2	Citric acid C_6_H_8_O_7_	191	1.462	+	+	+
**Phenolic acid**						
3	Quinic acid C_7_H12O_6_	191	0.958	+	+	−
**Total flavonoids**						
4	(e) Gallocatechin‐(e) Gallocatechin C_30_H_26_O_14_	609	6.926	+	−	+
5	(e) Catechin‐ (e) Gallocatechin C_30_H_26_O_13_	593	8.037	+	−	+
6	(e) Catechin‐(e) Catechin C_30_H_26_O_12_	577	5.729	+	−	+
7	Epigallocatechin C_15_H_14_O_7_	305	8.473	+	−	+
8	Catechin C_15_H_14_O_6_	289	8.652	+	−	+
9	Quercetin‐3‐sophoroside‐7‐rhamnoside C_39_H_50_O_26_	771	5.833	+	+	+
11	Kaempferol‐3‐*O*‐sophoroside‐7‐*O*‐rhamnoside C_33_H_40_O_20_	755	6.016	+	+	+
12	Isorhamnetin‐3‐*O*‐sophoroside‐7‐*O*‐rhamnoside C_34_H_42_O_21_	785	7.243	+	+	+
13	Kaempferol‐3‐glucoside‐7‐rhamnoside C_27_H_30_O_15_	593	8.147	+	+	+
14	Rutin C_27_H_30_O_16_	609	6.94	+	+	+
15	Isorhamnetin‐ glucoside‐rhamnoside derivate C_28_H_32_O_16_	623	6.588	+	+	+
16	Narcissin C_28_H_32_O_16_	623	7.29	+	+	+
17	Isorhamnetin‐3‐glucoside C_22_H_22_O_12_	477	7.789	+	+	+
18	Isorhamnetin C_16_H_12_O_7_	315	8.884	+	+	+

Among the biologically active compounds in preparations obtained from sea buckthorn fruit and processing products, 18 compounds were identified: 2 organic acids (malic acid, citric acid), 1 phenolic acid (quinic acid), 15 flavonoids [(e) gallocatechin‐(e) gallocatechin, (e) catechin‐ (e) gallocatechin, (e) catechin‐(e) catechin, epigallocatechin, catechin, quercetin‐3‐sophoroside‐7‐rhamnoside, kaempferol‐3‐O‐sophoroside‐7‐O‐rhamnoside, isorhamnetin‐3‐O‐sophoroside‐7‐O‐rhamnoside, kaempferol‐3‐glucoside‐7‐rhamnoside, rutin, isorhamnetin‐ glucoside‐rhamnoside derivate, narcissin, isorhamnetin‐3‐glucoside, isorhamnetin].

#### 3.1.1 Quantitative Analysis of Phenolic Compounds and Carotenoids

The spectral method was used to determine the content of total phenols, phenolic acids, total flavonoids, catechins, and leucoanthocyanins in sea buckthorn berries collected in different regions of Georgia. Antioxidant activity was also determined. Comparing the obtained results, it can be noted that the content of total phenols was 19.90–23.96 mg/g dry weight in samples taken from the territory of Adjara, and relatively higher in samples from Samtskhe‐Javakheti and Imereti (30.58–34.94 mg/g).

**FIGURE 2:**
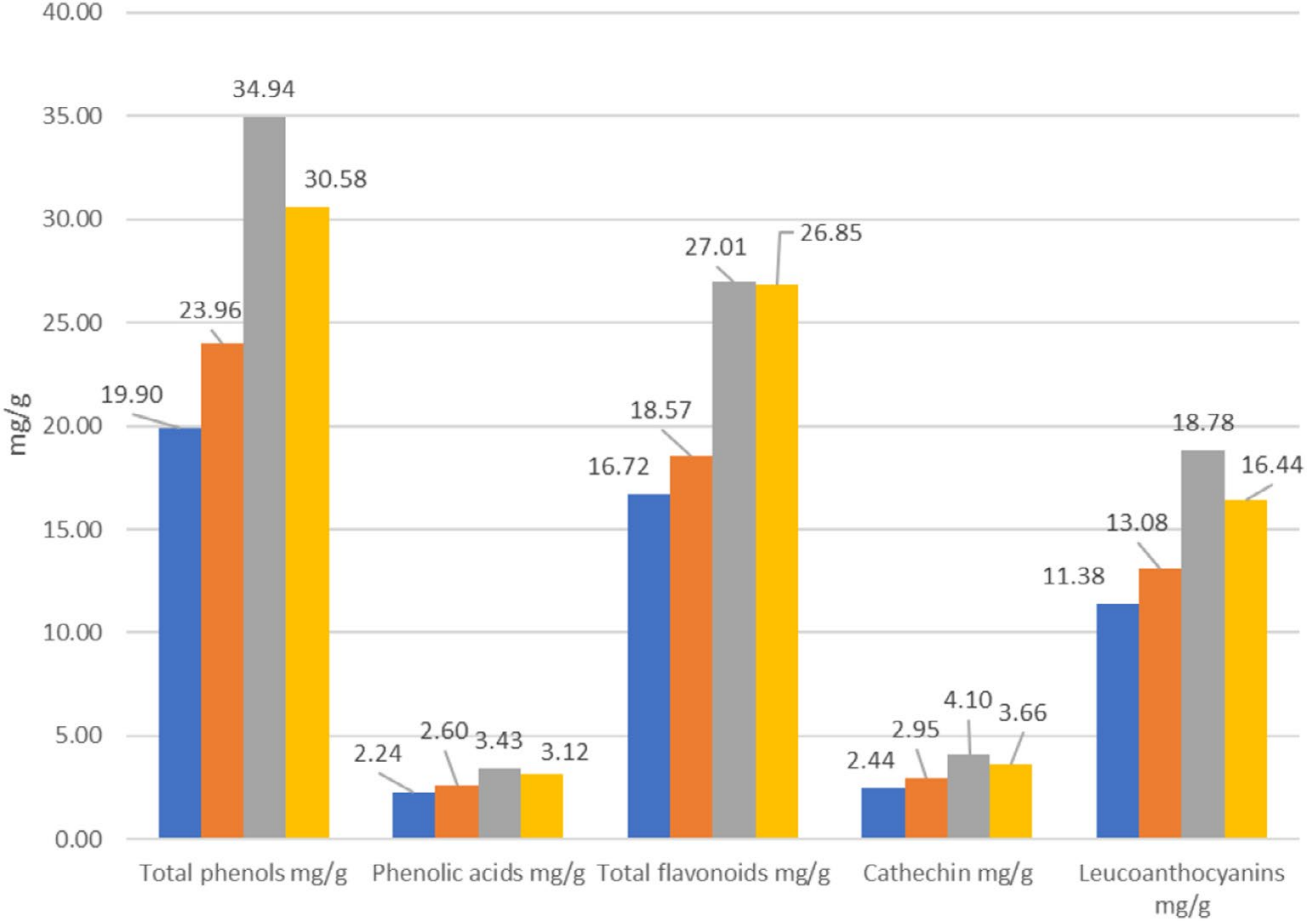
Phenol content in sea Buckthorn fruits collected in different regions of Georgia.

A similar ratio is observed for phenolic acids (2.24–2.6 and 3.12–3.43 mg/g) and flavonoids (16.72–18.57 and 26.85–27.01 mg/g). The content of catechins ranges from 2.44 to 4.10 mg/g, leucoanthocyanins—from 11.38 to 18.78 mg/g (Figure 2).

The total carotenoid content in sea buckthorn is 0.361–3.363 mg/g, with the highest content recorded in samples from Samtskhe‐Javakheti (3.363 mg/g) and Imereti (3.051 mg/g) (calculated on a dry weight basis) (Table 2).

**TABLE 2:** Carotenoid content in sea buckthorn fruits collected in different regions of Georgia.

Carotenoid content in sea buckthorn fruits, calculated as mg/g dry weight			
Region	Village	C mg/g raw weight	C mg/g dry weight
Adjara	Erge	0.604 ± 0.021	2.617 ± 0.034
Adjara	Tkhilnari	0.361 ± 0.01	1.564 ± 0.021
Samtskhe‐Javakheti	Akhaltsikhe	0.704 ± 0.013	3.051 ± 0.035
Imereti	Terjola	0.776 ± 0.025	3.363 ± 0.033

#### 3.1.2 Sea Buckthorn Fruit Processing

**FIGURE 3:**
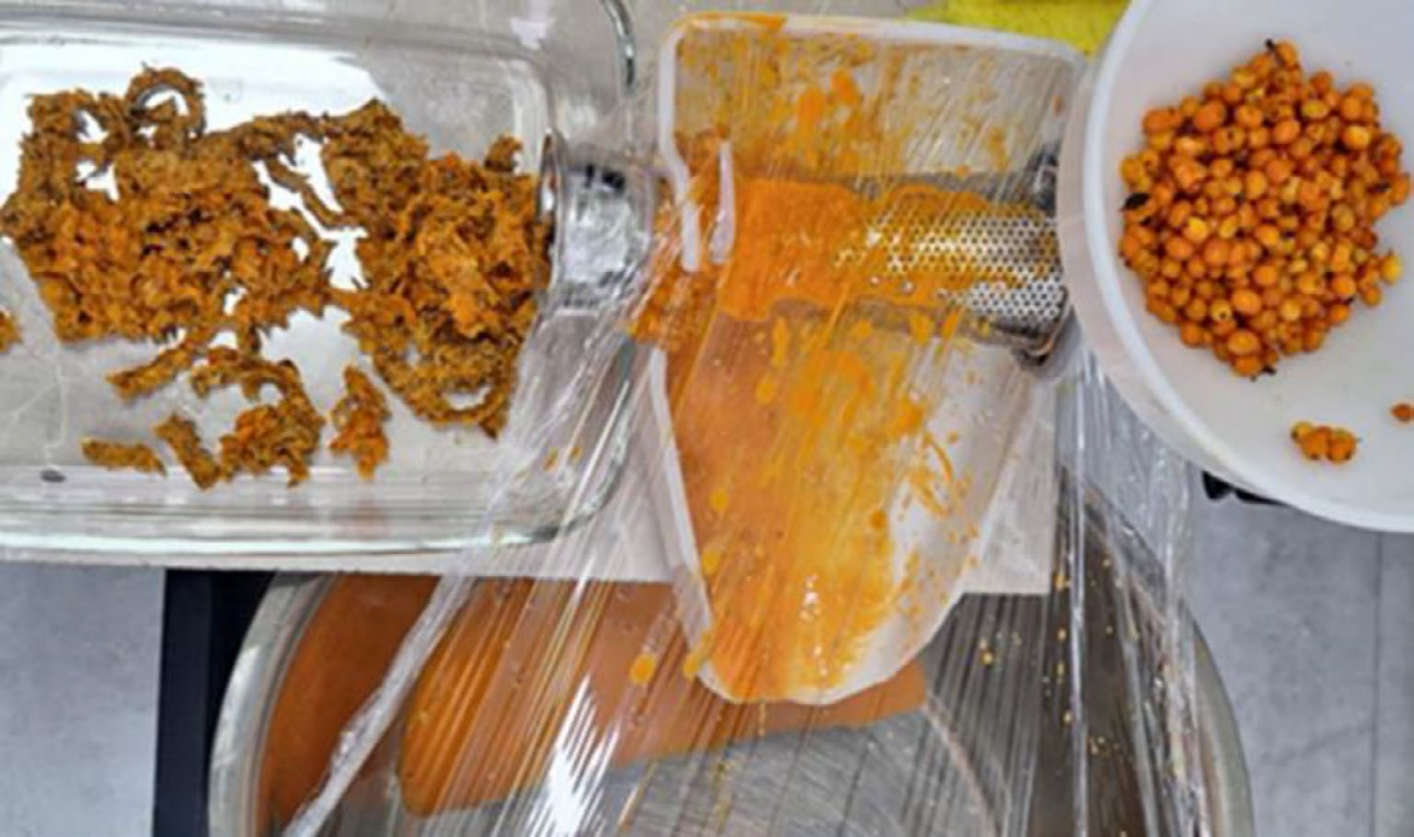
Berry grinding process: 1—pulp fraction and 2—the remaining solids (seeds and berry skin).

4500 g of sea buckthorn berries (raw) were taken for processing after grinding 2 fractions were obtained: pulp weight—3900 g (87% of the total erries' weight), which mainly consists of mesocarp and endocarp, and pressed pulp—600 g (13% of the total berries' weight). It mainly contains the skin and seeds of the berries (Figure 3).

**FIGURE 4:**
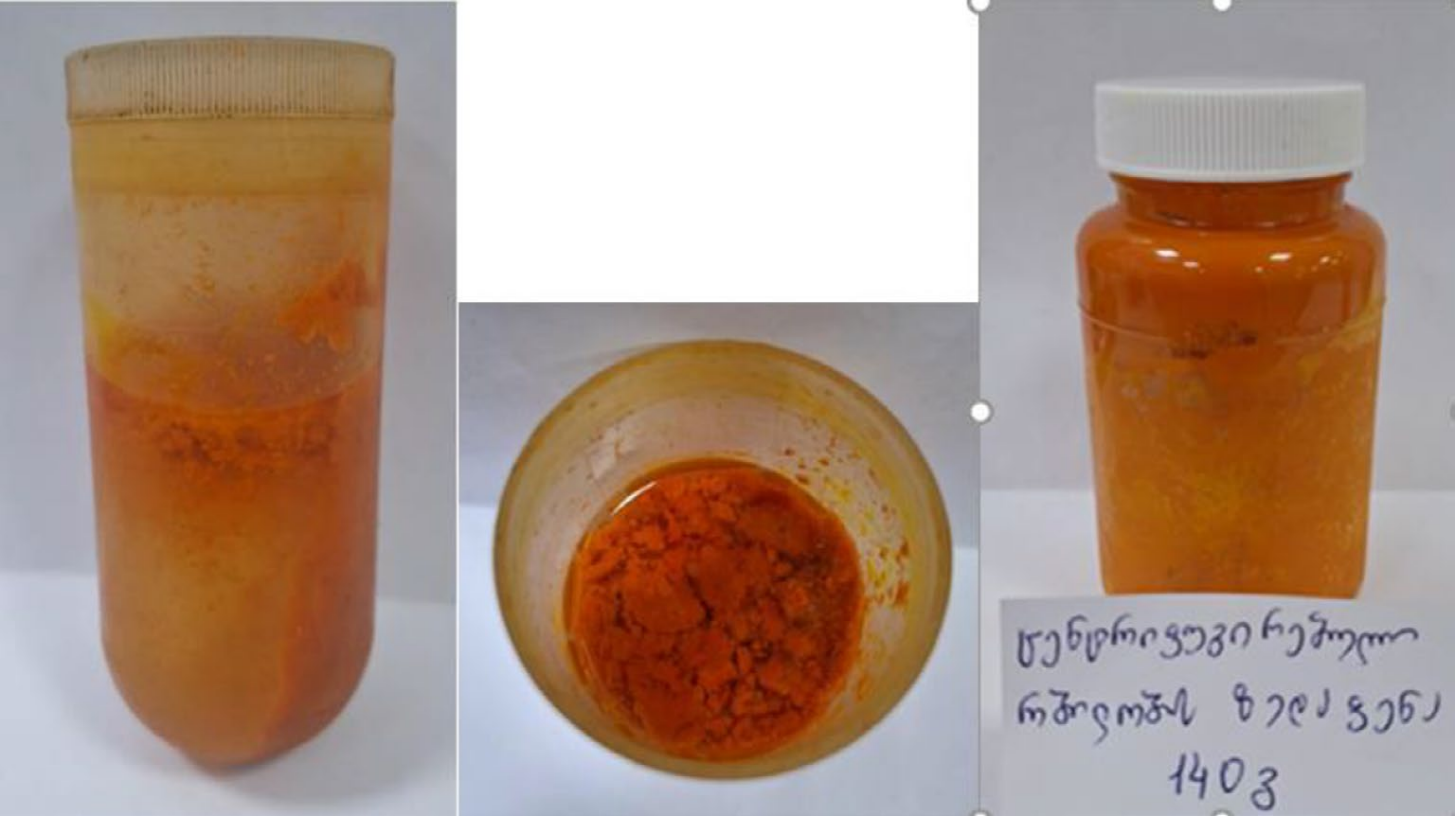
The upper part of the centrifuged mass of the pulp fraction is the arotenoids fraction (140 g) suspended in lipids.

**FIGURE 5:**
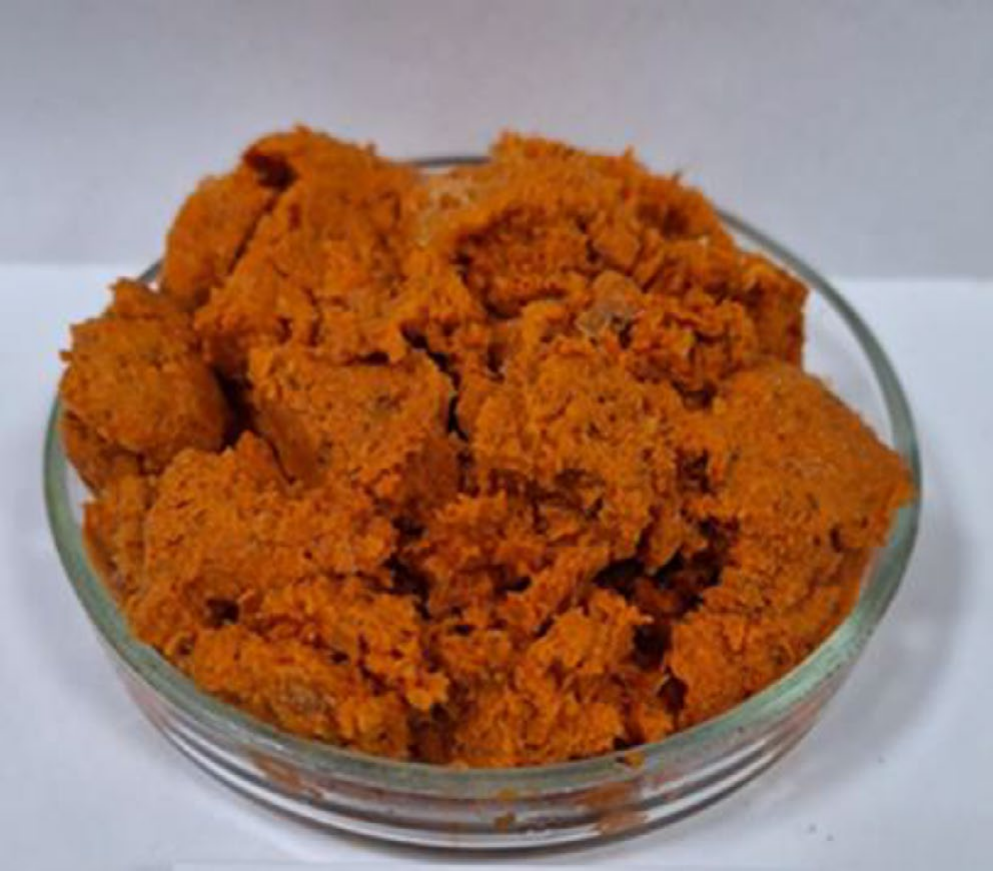
The bottom layer obtained from centrifugation of the pulp fraction is 1060 g, which is 27.17% of the pulp fraction.

For further separation and fractionation of the pulp, we centrifuged this mass for 2 min at a temperature of 20°C and parameters of 12,000 revolutions/s. As a result of centrifugation of the pulp fraction, 3 layers were separated: 1—the upper layer, which is mainly the carotenoids fraction suspended in lipids (140 g). It makes up 3.59% of the total pulp mass. The second, middle layer is juice (2700 g), which makes up 69.23% of the total pulp mass, and the third layer is the centrifuged sediment, 1060 g (27.17%) of the pulp mass (Figures 4 and 5 and Tables 3 and 4).

**TABLE 3:** Processing of raw sea buckthorn fruits—fractionation in a grinding machine.

Processing of raw sea buckthorn fruits—fractionation in a grinding machine				
Sample	Fractions by % yield	Carotenoid content		Carotenoid content, taking into account the yield of each fraction C mg/g dry weight
		C mg/g of raw weight	C mg/g of dry weight	
Berries	100	0.704 ± 0.02	3.051 ± 0.031	3.051 ± 0.031
Pulp	87	0.728 ± 0.018	3.155 ± 0.033	2.745 ± 0.028
Raw pressed—berry skins and seeds	13	1.431 ± 0.024	1.817 ± 0.022	0.236 ± 0.01

As a result of the quantitative analysis of carotenoids, it was determined that their content in raw sea buckthorn berries amounts to 3.051 mg/g on a dry weight basis. The majority of carotenoids (2.745 mg/g) are concentrated in the pulp fraction, while the content in the press cake fraction is only 0.236 mg/g (Table 3). Nearly two‐thirds of the carotenoids present in the pulp (1.817 mg/g) are found in the upper centrifuged layer, which corresponds to the lipophilic fraction co‐extracted with fat (Preparation 1). The fat content in this preparation represents approximately 3% of the fresh berry mass. Only a minor portion of the carotenoids (0.046 mg/g) remains in the juice, whereas 0.868 mg/g is retained in the centrifuged pulp mass (Table 4).

**TABLE 4:** % yield and content of carotenoids in fractions obtained by centrifugation of the pulp fraction.

Fraction I—Pulp	Fractions by % yield	Carotenoid content	
		C mg/g of raw weight	C mg/g dry weight
Centrifugation—top layer—carotenoids and lipids	3.58 ± 0.038	1.431 ± 0.022	1.817 ± 0.03
Centrifuged—middle layer—juice	69.23 ± 0.354	0.004 ± 0.0008	0.046 ± 0.0009
Centrifugation—bottom layer—sediment with pulp	27.17 ± 0.235	0.205 ± 0.004	0.868 ± 0.01

#### 3.1.3 Ultrasonic Extraction

For maximum extraction and separation of carotenoids from the centrifuged pulp mass, ultrasonic extraction was used using a “green” extractant, namely, pulp extraction with vegetable lipids and ethanol of various concentrations using an ultrasonic probe.

To select the optimal extraction conditions, individual parameters such as solid mass to extractant ratio, extraction temperature, time, and extractant were determined.

**FIGURE 6:**
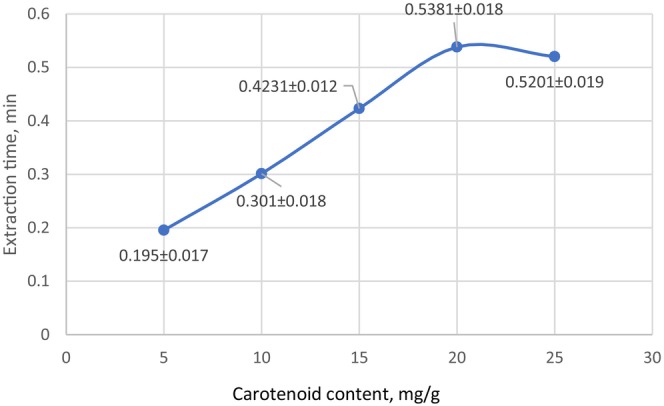
Curve showing the extraction of carotenoids from centrifuged sea buckthorn pulp using an ultrasonic probe.

Optimal conditions for vegetable lipids extraction using an ultrasonic probe were selected: the ratio of solid mass (centrifuged oilseed pulp—raw mass) and sunflower oil was 1:1, the temperature was 30°C–35°C (the temperature was controlled using an ice bath), the amplitude was 50%, and the extraction duration was 20 min. The extraction efficiency was determined by scanning the extracted carotenoids in the lipids in the ultraviolet and visible range every 5 min of extraction. During the first 20 min of extraction, the carotenoid content increased from 0.1954 to 0.5381, and during the next 5 min, no further increase in the pigment concentration was observed (Figure 6).

In carotenoid‐enriched and original sunflower oil samples, we determined the content of free fatty acids, peroxide value, anisidine index, carotenoid content, and antioxidant activity. Compared with the control sample of sunflower oil, the acidity is stable, the peroxide and anisidine values have decreased, namely, the acidity index is 0.05% (control oil) and 0.06% (enriched oil). The peroxide value is from 2.8 to 2.56, the anisidine value is from 3.73 to 3.22. This indicates that the amount of aldehyde compounds and peroxides in the oil enriched with carotenoids is lower compared with the control sample of oil, which increases the oxidative stability of the oil (Table 5).

**TABLE 5:** Quality indicators of sunflower seeds enriched with carotenoids.

Quality indicators	A control sample of sunflower oil	Sunflower oil enriched with carotenoids
Free fatty acid (as Oleic acid, MW 282) %	0.05 ± 0.003	0.06 ± 0.003
Peroxide Value	2.80 ± 0.047	2.56 ± 0.03
Anisidine value	3.73 ± 0.041	3.22 ± 0.04
Carotenoids %	—	0.059 ± 0.001
Antioxidant activity, 50% inhibition of DPPH radical, 0.1 mmol/L per mg sample	1.17 ± 0.018	0.33 ± 0.005

As a result of determining the antioxidant activity, it was found that the oil enriched with carotenoids is characterized by 3.5 times higher antioxidant activity compared to the control sample. In particular, 0.33 mg of carotenoid‐enriched oil was required for 50% inhibition of the DPPH radical at a concentration of 0.1 mmol/L, while 1.17 mg was required for the control oil. The results indicate that carotenoids extracted from sea buckthorn pulp into oil (on average 0.059% dry weight) significantly increase the antioxidant activity of the oil (Table 5).

At the next stage of sea buckthorn fruit processing, the research aimed to determine the optimal conditions for maximum extraction of carotenoids and other biologically active phenolic compounds from centrifuged sea buckthorn pulp using green solvents. For this purpose, extraction using an ultrasonic probe was used. At the initial stage, the efficiency of the extractant was tested, namely, water, 25%, 50%, and 75% ethanol. The ratio of the solid mass and the extractant was also determined, the optimal ratio being 1:20. The extracted mass, together with the extractant, was homogenized at 7000 rpm for 1 min. Four temperature modes were also selected for extraction: 30°C–35°C, 35°C–40°C, 40°C–45°C, and 45°C–50°C. At the first stage, the optimal type of extractant was determined based on the content of extractable substances, antioxidant activity, carotenoids, and the sum of phenolic compounds. With an increase in the concentration of ethanol, the content of extractable substances does not increase, but the amount of carotenoids and phenols increases, due in which the antioxidant activity is also high. The highest results were obtained using 75% ethanol, where the concentration of carotenoids was 1.684 mg/g, and phenols—8.213 mg/g. The carotenoid content is almost two times less in the 50% ethanol extract (0.703 mg/g) and significantly less in the 25% ethanol (0.282 mg/g) and aqueous extracts (0.121 mg/g). The content of total phenols increases in parallel with the ethanol concentration (from 2324 to 8213 mg/g) (Table 7). Accordingly, the antioxidant activity characteristic is also high: 0.163 mg of sample is sufficient for 50% inhibition of the DPPH radical (in the case of 75% ethanol extract), whereas in the case of other extracts, the sample mass required for inhibition increases from 0.239 to 0.547 mg. The data obtained indicate that 75% ethanol is the optimal extractant for obtaining biologically active compounds from sea buckthorn pulp (Table 6).

**TABLE 6:** Selection of extraction conditions for biologically active compounds from sea buckthorn pulp using the ultrasonic probe method.

Extragent	Extractive substances %	Carotenoids in mg/g dry weight	Extracted phenols in mg/g by weight	Antioxidant activity—50% inhibition of DPPH radical per mg sample
Water	6.61 ± 0.111	0.121 ± 0.015	2.324 ± 0.142	0.647 ± 0.074
25% Ethanol	7.31 ± 0.121	0.282 ± 0.01	3.805 ± 0.073	0.470 ± 0.014
50% Ethanol	7.29 ± 0.081	0.703 ± 0.02	6.661 ± 0.152	0.239 ± 0.019
75% Ethanol	7.21 ± 0.123	1.684 ± 0.054	8.213 ± 0.147	0.163 ± 0.011

**FIGURE 7:**
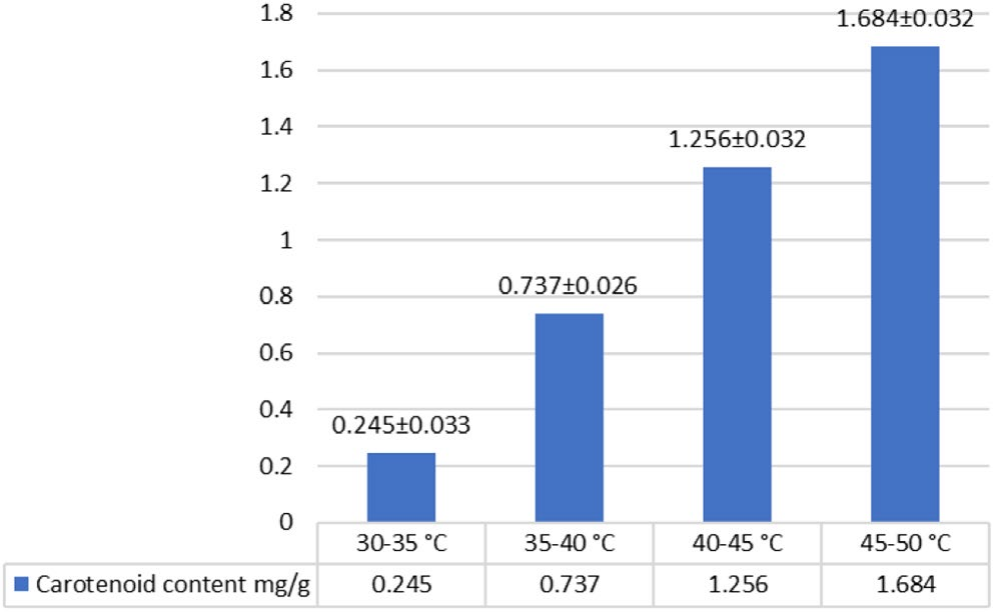
Determining the optimal temperature conditions for carotenoid extraction using an ultrasonic probe.

Taking into account the thermolability of carotenoids, experiments were conducted at various temperature conditions to determine the optimal extraction temperature. As a result, it was found that the maximum extraction of carotenoids is achieved at a temperature of 45°C–50°C. With an increase in temperature, the extraction of carotenoids increased significantly from 0.245 mg/g (30°C–35°C) to 1.684 mg/g (45°C–50°C) (Figure 7).

**FIGURE 8:**
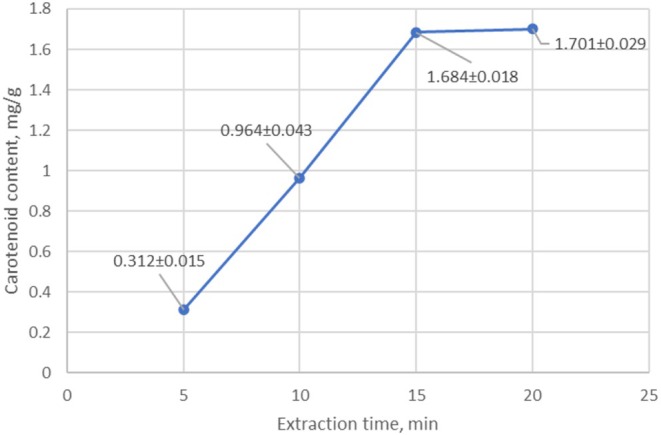
Determining the optimal time for carotenoid extraction using an ultrasonic probe.

To determine the optimal extraction conditions, the effect of temperature and time on extraction efficiency was determined. Experiments were conducted at different time intervals to optimize extraction time. As a result, it was found that the maximum yield of carotenoids was achieved with 15‐min ultrasonic treatment (Figure 8). During the next 5 min, the amount of carotenoids did not increase. As a result, it was found that ultrasonic extraction with 75% ethanol at a temperature of 45°C–50°C for 15 min provides the maximum yield of carotenoids (Figure 8). Longer extraction does not lead to a significant increase in carotenoid content, which indicates that the extraction process is complete.

**FIGURE 9:**
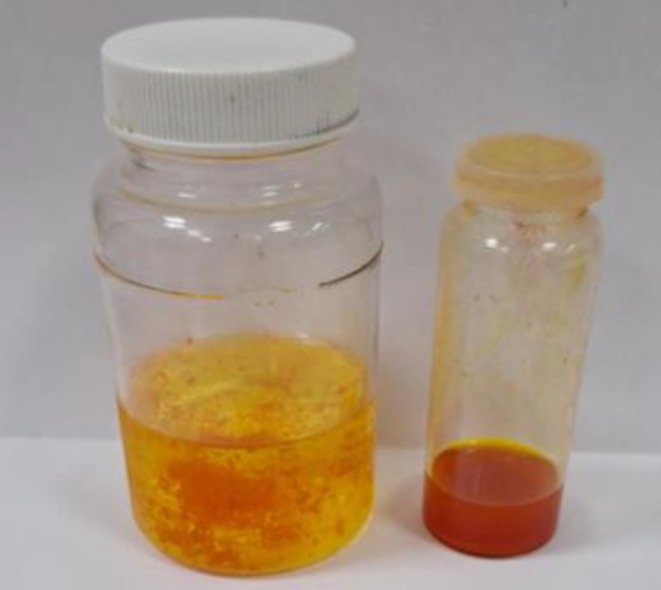
The mass of carotenoids obtained by precipitation from the ethanolic extract at 4°C.

To isolate biologically active compounds from the obtained extract, a fractionation process was carried out. In particular, to isolate carotenoids, the extract was cooled to 4°C for 24 h, which led to the precipitation of carotenoids (Figure 9). To separate the resulting sediment, the extract was centrifuged at 12,000 rpm for 2 min. The resulting sediment containing carotenoids was lyophilized to obtain a preparation containing carotenoids. To obtain phenolic compounds, the supernatant obtained after centrifugation was concentrated in a rotary vacuum apparatus at a temperature of 40°C. The concentrate was lyophilized to obtain a powder containing a phenolic compound.

#### 3.1.4 Processing of Residue

**FIGURE 10:**
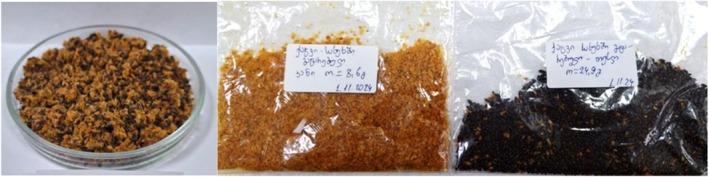
Comprising 13% of the total berry mass, the press cake fraction is composed of 20% berry skin and 80% seeds.

The press cake remaining after sea buckthorn juice production represents a significant source of lipids and biologically active compounds, including phenolic compounds (Table 7). According to our research findings, raw sea buckthorn press cake, which consists of approximately 80% seeds and 20% fruit skin, contains 11.95%–13.22% lipids (calculated on a dry weight basis) and 0.140–0.583 mg/g carotenoids (Figure 10).

**TABLE 7:** Pressing—mechanical separation of berry skins and seed fraction.

Pressing—mechanical separation of berry skins and seed fraction (using a sieve of 0.5–1.0 mm)				
Fraction II—Pressing	Fractions by % yield	Carotenoid content		Carotenoid content, taking into account the yield of each fraction, C mg/g dry weight
		C mg/g of raw weight	C mg/g of dry weight	
Seed	80	0.130 ± 0.014	0.140 ± 0.019	0.112 ± 0.003
Berry skins	20	0.566 ± 0.006	0.583 ± 0.015	0.117 ± 0.003

Quantitative analysis of carotenoids shows that the carotenoid content in individual fractions is almost the same: 0.112 mg/g in seeds (based on 80% yield) and 0.117 mg/g in the peel (based on 20% yield) (dry weight) (Table 7).

Spectral analysis of phenolic compounds shows that the total phenol content in raw pulp is 89.41 mg/g, of which 16.95 mg/g are phenolic acids and 64.71 mg/g are flavonoids. Flavonoids are mainly represented by catechins (25.90 mg/g) and leucoanthocyanins (44.45 mg/g). The antioxidant activity of sea buckthorn extract is quite high: 0.766 mg of sea buckthorn extract is sufficient to inhibit 50% of DPPH radicals.

Based on the results obtained, our goal was to minimize the waste of sea buckthorn pressing and maximize the use of its valuable components through green technologies. For this purpose, it was used ultrasonic probe extraction and supercritical water extraction were used, which are characterized by high efficiency, selectivity, and ecological purity.

**FIGURE 11:**
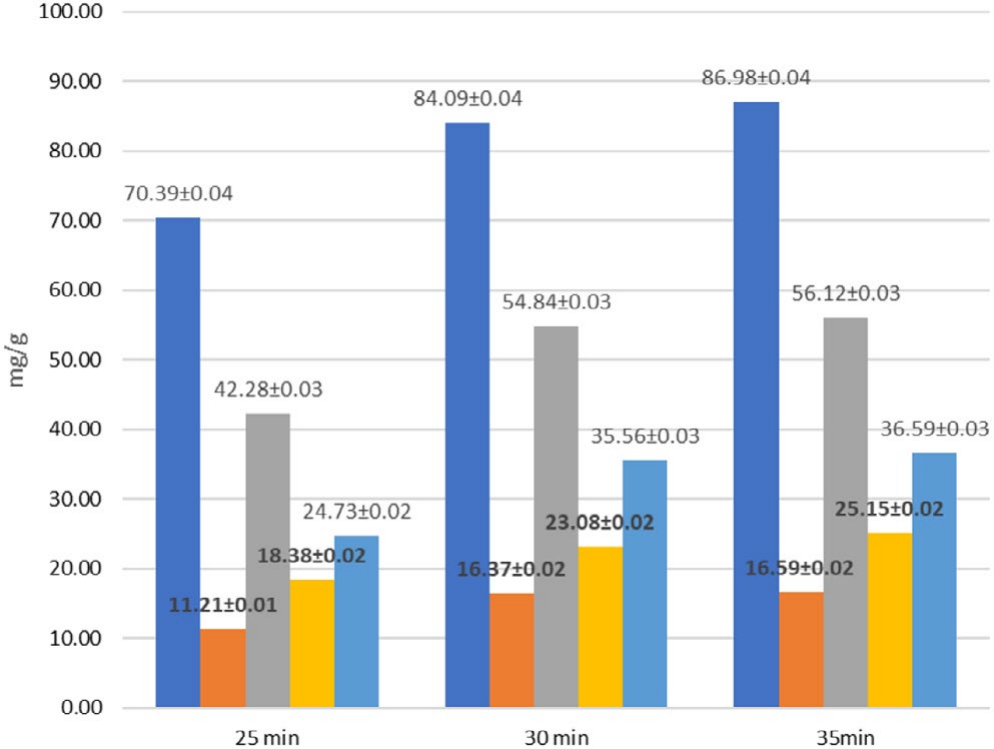
Extraction of biologically active compounds from raw sea buckthorn pulp using an ultrasonic probe.

**FIGURE 12:**
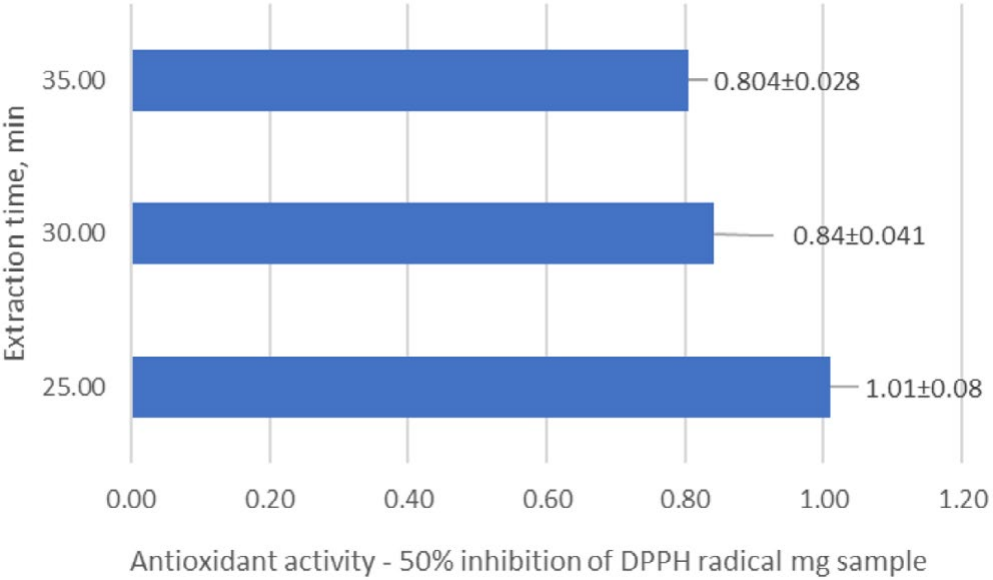
Antioxidant activity‐extraction of sea buckthorn crude extract using an ultrasonic probe.

The efficiency of the experiment was determined by the extraction yield, which was calculated based on the content of lipids, carotenoids, and phenolic compounds. At the initial stage, the sample was homogenized with the extractant for 2 min to improve the pulp quality and hydration (15,000 rpm—the number of revolutions per minute, WITEG homogenizer). The optimum extraction ratio was 1:20 solid to liquid, 50% amplitude, pulse duty cycle, and 50% ethanol solvent. After 25 min of extraction, the total phenols were 70.39 mg/g, phenolic acids 11.21 mg/g, flavonoids 42.28 mg/g, catechins 18.38 mg/g, and leucoanthocyanins 24.73 mg/g (dry weight basis) (Figure 11). During the next 5, 30 min after extraction, the content of these compounds increased from 4 to 12 units (phenols from 70.39 to 84.09) (Figure 11). A further increase in the extraction time did not lead to a significant increase in the extraction yield, so 30 min was considered the optimal time. In the next step, we centrifuged the extract at 7000 rpm for 2 min at 20°C. The resulting extract was left at room temperature (24 h), and the lipids dissolved. The amount of lipids obtained was 7.365%, which is approximately 62% of the theoretical lipids yield (compared to the value obtained by the classical Soxhlet method; 11.95%) (Table 8). The antioxidant activity of the extracts was determined in parallel, and a correlation was found between the concentration of phenolic compounds and the antioxidant activity (Figure 12).

**TABLE 8:** Biologically active compounds and antioxidant activity in Sea Buckthorn extract (Seeds/Skin).

Biologically active compounds and antioxidant activity	*Sea buckthorn* Extract (Seeds/Skin)	
	Raw	Dried in a vacuum
Total phenols mg/g	91.67 ± 0.241	89.41 ± 0.230
Phenolic acids mg/g	18.38 ± 0.188	16.95 ± 0.111
Total flavonoids mg/g	70.88 ± 0.208	64.71 ± 0.145
Catechins mg/g	29.86 ± 0.229	25.90 ± 0.217
Leucoanthocanins mg/g	40.56 ± 0.327	44.45 ± 0.172
Antioxidant activity—50% inhibition of DPPH radical per mg sample	0.638 ± 0.008	0.766 ± 0.01

After removal of lipids, the extract was concentrated under a vacuum at a temperature of 45°C, and the resulting concentrate was lyophilized.

#### 3.1.5 The Supercritical Water Extraction

The mass of the resulting preparation was determined during the supercritical water extraction process, and the solid sample‐to‐water ratio was maintained at 1:20, which corresponds to the ratio used in the previous experiment. The extraction efficiency was assessed by the yield of biologically active compounds.

**FIGURE 13:**
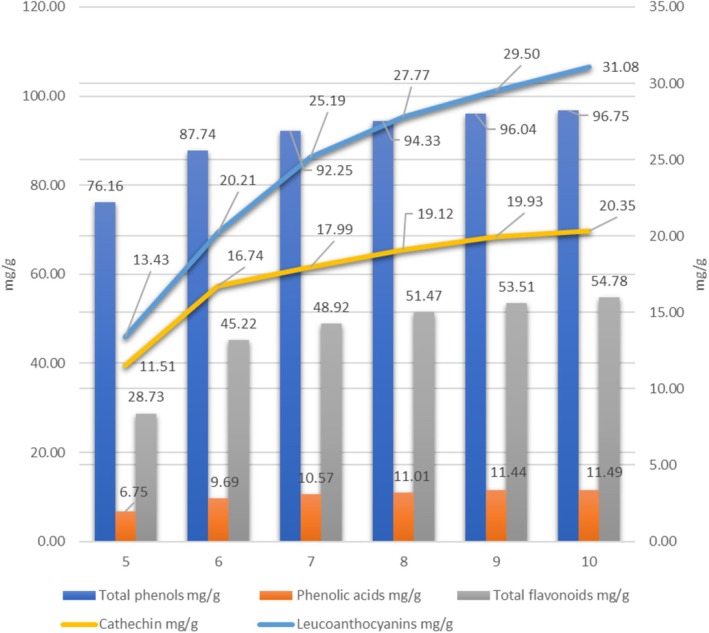
Supercritical water extraction conditions of sea buckthorn crude cake (seed/skin) and phenolic compound content.

In contrast to ultrasonic extraction, the total phenolic content in the extract obtained by supercritical water extraction for 5 min was 76.16 mg/g (phenolic acids—6.75 mg/g, flavonoids—28.73 mg/g, including catechins—11.51 mg/g and leucoanthocyanins—13.43 mg/g) (Figure 13). With an increase in the extraction time to 6 min, the total phenolic content increased to 87.74 mg/g, and after 7 min it reached 92.25 mg/g. The results show that the optimal extraction time with supercritical water to obtain the maximum yield of phenolic compounds is 7 min. During the next 3 min of extraction, the total phenolic content increased slightly, by about 5 units (from 92.25 to 96.75 mg/g) (Figure 13). The results show that supercritical water extraction for 7 min provides the maximum yield of phenolic compounds. After decanting the resulting aqueous extract, we left it at room temperature (24 h), and the lipids dissolved. The amount of lipids obtained was 10.27%, which is approximately 86% of the theoretical lipids yield compared to the value obtained by the classical Soxhlet method (11.95%) (Table 9).

**TABLE 9:** Lipid content in sea buckthorn pulp using different methods.

Lipid content in sea buckthorn pulp using different methods			
Samples	Classic method—hexane extraction	Extraction with an ultrasonic probe	Supercritical water extraction
Seeds/skin	11.95 ± 0.214	7.365 ± 0.231	10.27 ± 0.218
Seeds	13.22 ± 0.261	8.097 ± 0.157	11.73 ± 0.222

However, in the lipids obtained by the supercritical water extraction method, the content of carotenoids was not detected, whereas in the lipids extracted using the ultrasonic probe, it was 0.105 mg/g in terms of dry weight. At the next stage, the aqueous extract was concentrated under vacuum (at a temperature of 55°C).

**FIGURE 14:**
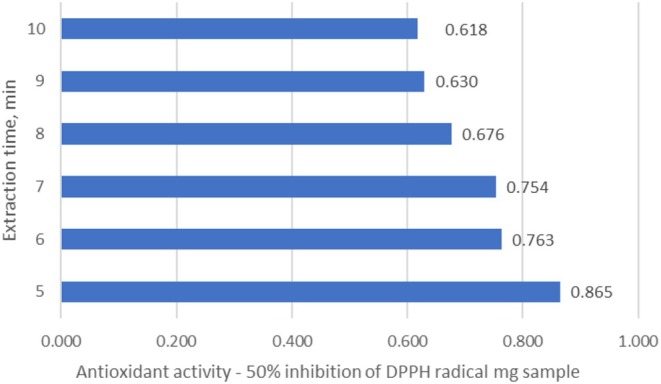
Antioxidant activity—50% inhibition of DPPH radical, mg sample—The supercritical water extraction of Sea buckthorn raw pulp (seeds/skin).

The results show that supercritical water extraction for 7 min provides the maximum yield of phenolic compounds (Figure 13). In parallel, the antioxidant activity of the extracts was determined, and a correlation was found between the concentration of phenolic compounds and the antioxidant activity (Figure 14).

#### 3.1.6 Lipid Compounds of Sea Buckthorn Fruits

**FIGURE 15:**
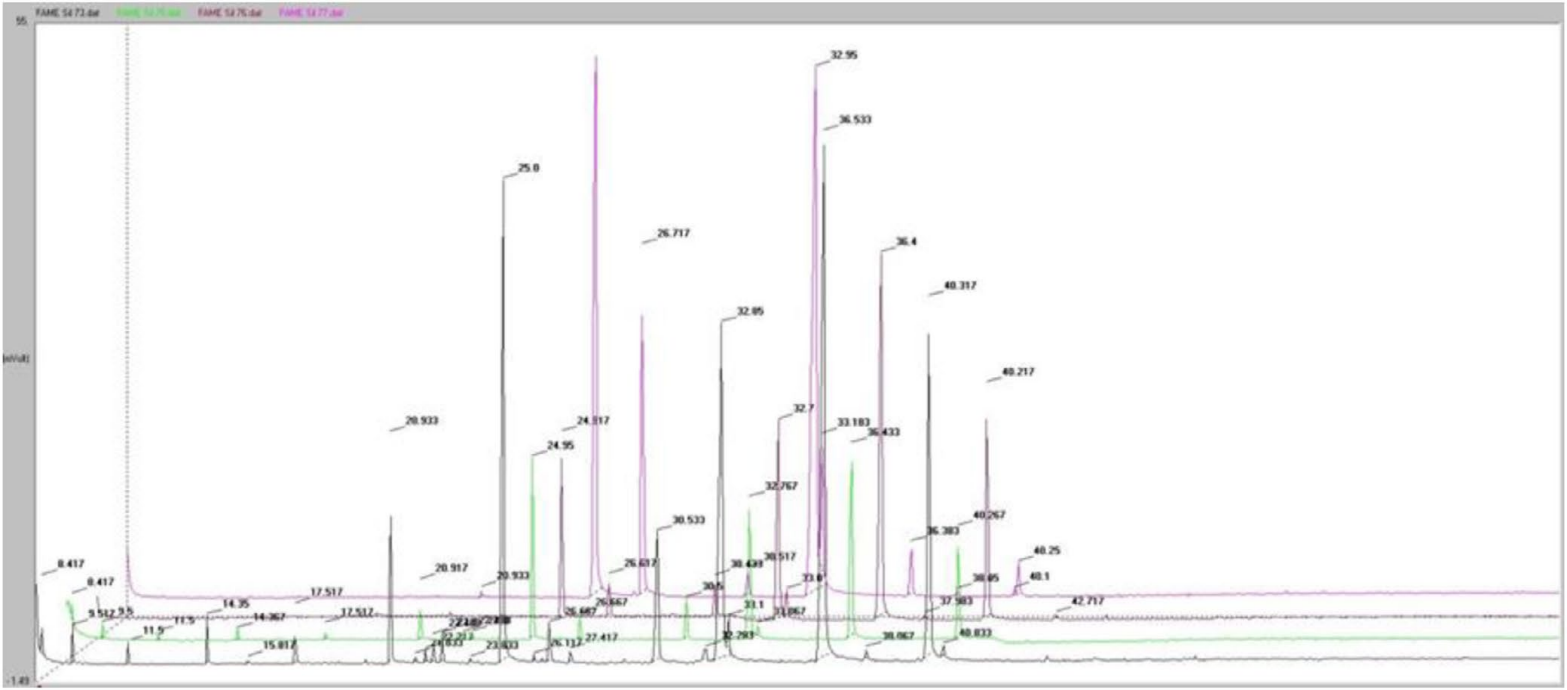
Comparison of chromatographic analysis of sea buckthorn oil.

For gas–liquid chromatography, the sample must be esterified, for which it is pre‐filtered to remove mechanical impurities (Figure 15). Then, 1 mL was transferred to a centrifuge tube, and 0.5 mL of 2 normal KOH 96% alcohol solution was added (ethanol or methanol can be used).

Then, 10 mL of hexane was added (total volume 11.5 mL). The mixture was vortexed until fully dissolved (at least 30 s) and centrifuged for 1 min at 13,500 rpm. 1.0 μL of the test sample from the upper fraction of the sample was injected using a 10 μL micro syringe from SGE Analytical Science.

Identification of the chromatographically obtained components was performed using a standard sample of known composition, Supelco 37 Component FAME Mix (product number: CRM47885, lot number: LRAD3869).

The results presented in the Table demonstrate the difference between the composition and concentration of fatty acids in lipids obtained by different methods.

Sea buckthorn seed oil obtained by the Soxhlet method is characterized by a variety of fatty acids containing 24 components, 5 of which are unknown (Table 10).

**TABLE 10:** Component composition of carboxylic acids.

№	Component name	Retention time (min)	Sea buckthorn seed oil obtained by Soxhlet extraction	Sea buckthorn seed and peel oil obtained by Soxhlet extraction	Sea Buckthorn seed oil sourced from the US	US, obtained from the seeds and skins of sea buckthorn	Sea Buckthorn seed oil and skin SWE
1	Butyric acid methyl ester (4:0)	8.417	1.038 ± 0.01	1.005 ± ±0.01	0	0	0
2	Caproic acid methyl ester (6:0)	9.750	0.612 ± 0.01	0.817 ± 0.01	0	0.413 ± 0.005	0. 764 ± 0.01
3	Caprylic acid methyl ester (8:0)	11.500	0.337 ± 0.005	0.361 ± 0.004	0	0	0
4	Capric acid methyl ester (10:0)	14.350	0.747 ± 0.01	0.853 ± 0.01	0	0	0
5	Undecanoic acid methyl ester (C11:0)	15.817	0.069 ± 0.001	0	0	0	0
6	Lauric acid methyl ester (C12:0)	20.933	0.966 ± 0.01	0.836 ± 0.01	0	0	0
7	Myristic acid methyl ester (C14:0)	20.917	4.371 ± 0.04	4.238 ± 0.04	0.090 ± 0.001	0	0
8	Unknown	21.833	0.086 ± 0.001	0	0	0	0
9	Unknown	22.217	0.203 ± 0.004	0	0	0	0
10	Myristic acid methyl ester (C14:1)	22.483	0.471 ± 0.005	0.429 ± 0.005	0	0	0
11	Pentadecanoic acid methyl ester (C15:0)	22.800	0.506 ± 0.01	0.417 ± 0.005	0	0	0
12	Unknown	23.833	0.064 ± 0.001	0	0	0	0
13	Palmitic acid methyl ester (C16:0)	25.000	16.317 ± 0.04	18.212 ± 0.04	10.164 ± 0.01	7.430 ± 0.01	10.121 ± 0.01
14	Unknown	26.117	0.105 ± 0.001	0	0	0	0
15	Palmitoleic acid methyl ester (C16:1)	26.667	1.024 ± 0.01	2.279 ± 0.02	2.121 ± 0.01	0.423 ± 0.005	1.605 ± 0.01
16	Heptadecanoic acid methyl ester (C17:0)	27.417	0.280 ± 0.003	0	0	0	0
17	Stearic acid methyl ester (C18:0)	30.500	5.808 ± 0.04	5.001 ± 0.03	2.948 ± 0.01	2.929 ± 0.01	3.038 ± 0.01
18	Elaidic acid methyl ester (C18:1n9t)	32.283	0.537 ± 0.01	0	0	0	0
19	Oleic acid methyl ester (C18:1n9c)	32.800	20.359 ± 0.04	21.552 ± 0.04	21.377 ± 0.03	17.697 ± 0.02	25.498 ± 0.03
20	Unknown	33.083	0.960 ± 0.01	1.267 ± 0.01	1.558 ± 0.01	1.059 ± 0.01	1.116 ± 0.01
21	Linoleic acid methyl ester (C18:2n6c)	36.400	30.724 ± 0.04	29.425 ± 0.04	46.512 ± 0.04	50.010 ± 0.04	48.335
22	Arachidic acid methyl ester (C20:0)	38.067	0.228 ± 0.005	0	0.229 ± 0.005	0.152 ± 0.005	0
24	α‐Linolenic acid methyl ester (C18:3n3c)	40.300	13.871	13.309 ± 0.03	14.740 ± 0.02	19.888 ± 0.02	9.331 ± 0.01
25	Unknown	40.833	0.317	0	0	0	0
26	*cis*‐11,14‐Eicosadienoic acid methyl ester (C20:2)	42.800	0	0	0.127 ± 0.005	0	0
27	Behenic acid methyl ester (C22:0)	44.567	0	0	0.133 ± 0.005	0	0.192 ± 0.005

The lipids obtained by the Soxhlet method are rich in low‐order fatty acids (C4:0)–(C15:0). They contain a large amount of saturated fatty acids, amounting to 31.740%. In combination with other methods (US and SWE), it ranges from 10.92% to 14.14%. The Soxhlet method also determines the content of trans lipids, in particular elaidic acid methyl ester (C18:1n9t) (Table 10).

This is an undesirable component in the food industry. When lipids from sea buckthorn pulp (seeds and berry skins) are extracted using ultrasonic probing, α‐Linolenic acid methyl ester (C18:3n3c), the maximum increase in its concentration reaches 19.888%, while the minimum content is recorded in oil obtained by the supercritical water extraction method (SWE) and is only 9.331%.

Linoleic acid methyl ester (C18:2n6c)‘s content decreases when obtained by the Soxhlet extraction method (29.425%) and reaches a maximum when using the ultrasonic probing method (50.010%).

The oil obtained from sea buckthorn berries using the SWE method also contains a fairly high content of oleic acid methyl ester (C18:1n9c)—25.5%, while in products obtained using other methods, its content fluctuates between 17.7% and 21.8% (Table 10).

## 4 Conclusions

Comparing the obtained results, we can conclude that by mechanical processing, passing the berries through a properly selected grinder, without any heat treatment and the use of organic solvents, we obtained a fraction of carotenoids suspended in lipids. Regarding carotenoid content, the lipophilic fraction yielded 1.817 mg of carotenoids, representing 66% of the total carotenoids present in the sea buckthorn pulp. To obtain preparations rich in biologically active substances, ultrasonic probe extraction was employed. During the extraction of the pulp fraction obtained during the processing of berries with sunflower oil, using the ultrasound probing method, sunflower oil enriched with carotenoids was obtained. This carotenoid‐enriched sunflower oil, produced through an efficient and environmentally friendly method, demonstrates significant potential for applications in the food and nutraceutical industries. Its high carotenoid content suggests its suitability as a natural food additive, enhancing the nutritional value and antioxidant properties of various food products. Furthermore, the simplicity and scalability of the extraction process make it attractive for large‐scale production of carotenoid‐rich ingredients for dietary supplements and other nutraceutical applications. From the obtained quality characteristics, it can be concluded that the addition of carotenoids led to some improvement in the quality of the oil. The observed reductions in acidity and peroxide value suggest that carotenoids exhibit significant antioxidant activity, effectively slowing oil oxidation. The optimal conditions for ultrasound‐assisted extraction using vegetable oil were determined as follows: a solid‐to‐liquid ratio of 1:1 (sea buckthorn centrifuged pulp—fresh weight—to sunflower oil), a temperature of 30°C–35°C (maintained using an ice bath), an amplitude of 50%, and an extraction duration of 20 min. From the obtained quality characteristics, it is evident that the incorporation of carotenoids notably enhanced the oil's quality. Specifically, our observations revealed significant reductions in n‐anisidine and peroxide values compared to the control sunflower oil samples. While the acidity remained relatively stable (0.05% for the control oil and 0.06% for the carotenoids‐enriched oil), both peroxide and n‐anisidine values decreased. The peroxide value decreased from 2.8 to 2.56, and the n‐anisidine value decreased from 3.73 to 3.22. These results indicate that the carotenoids‐enriched oil exhibits lower levels of aldehyde compounds and peroxides compared to the control oil samples, thereby demonstrating enhanced oxidative stability.

This study effectively determined the optimal solvent composition for the ultrasonic‐assisted extraction of key bioactive compounds from centrifuged sea buckthorn pulp. Among the tested hydroethanolic mixtures, 75% ethanol demonstrated superior efficacy as an extractant. This condition yielded the highest concentrations of both carotenoids (1.684 mg/g) and total phenolic compounds (8.213 mg/g). While an increase in ethanol concentration did not proportionally elevate the total extractable mass, it selectively enhanced the solubility and recovery of the target lipophilic (carotenoids) and more polar (phenols) compounds. These findings underscore the suitability of 75% ethanol as the solvent of choice for maximizing the extraction of valuable lipophilic and phenolic antioxidants from sea buckthorn pulp using ultrasonic‐assisted methods.

This study rigorously compared two green extraction methodologies, ultrasonic probe extraction and supercritical water extraction (SWE), for the efficient recovery of phenolic compounds from sea buckthorn biomass. Ultrasonic‐assisted extraction of sea buckthorn pomace, optimized at a solid‐to‐liquid ratio of 1:20 with 50% ethanol as the solvent, 50% amplitude, and a 50% pulse duty cycle for 25 min, yielded a significant total phenolic content of 70.39 mg/g (dry weight), encompassing notable concentrations of phenolic acids, flavonoids (including catechins and leucoanthocyanins).

Concurrently, the SWE of sea buckthorn pulp demonstrated its effectiveness in extracting phenolic compounds, with the extraction time identified as a critical parameter. A maximum total phenolic yield of 92.25 mg/g was achieved after 7 min of extraction under a 1:20 solid‐to‐water ratio. While extending the extraction time offered minimal additional benefit, 7 min was established as the optimal duration for maximizing phenolic recovery under the investigated SWE conditions. Furthermore, SWE facilitated the concurrent separation of a substantial lipid fraction, achieving 86% recovery compared to conventional Soxhlet extraction, highlighting its potential for integrated multi‐component extraction.

Comparing the obtained data, the phenolic content in the preparation obtained from the raw pomace using the ultrasonic probe method was 71.4 mg/g dry weight, while it was 80 mg/g dry weight with supercritical water extraction. The antioxidant activity was almost similar in both cases; for 50% inhibition of the DPPH radical, 0.8 mg of the preparation was sufficient with ultrasonic extraction, and 0.754 mg of the preparation obtained with supercritical water extraction was sufficient.

The findings underscore the viability of both ultrasonic extraction and SWE as environmentally sustainable alternatives for valorizing sea buckthorn by‐products. While ultrasonic extraction with hydroethanolic solvents effectively recovers a broad spectrum of phenolics from pomace, SWE offers the advantages of shorter processing times and the use of a non‐toxic solvent (water) for extracting a high yield of phenolics from pulp, alongside the added benefit of partial lipid recovery. These characteristics position both methods as valuable tools for the production of bioactive‐rich extracts for applications across the food, cosmetic, and pharmaceutical industries, contributing to the development of natural and sustainable ingredients. Future research should focus on further process optimization, scalability assessments, and detailed characterization of the extracted compounds to fully realize their potential.

As a result of the experiment, all other parameters selected for extraction turned out to be highly optimal, which is confirmed by a qualitative and quantitative study of the active substances in the obtained preparation. In particular, all the compounds found in sea buckthorn fruits are present in both preparations. In the pressed preparation, we obtain flavonoids (e) Gallocatechin‐(e) Gallocatechin, (e) Catechin‐ (e) Gallocatechin, (e) Catechin‐(e) Catechin, Epigallocatechin, Catechin and quercetin, isorhamnetide and kaempferol derivatives, in pulp extract Quinic acid, Rutin, Narcissin, Quercetin, isorhamnetin and kaempferol derivatives. The preparation obtained from pulp treated with an ultrasonic probe is characterized by high antioxidant activity. In particular, 0.163 mg of dry pulp extract is sufficient for 50% inhibition of DPPH radical, while in the case of the preparation obtained by pressing, 0.618–0.8 mg of dry sample is sufficient. However, about 13% of the oil is also obtained by pressing, which gives pressing additional functions and value. Based on the results, it can be concluded that both sea buckthorn seeds and the entire fruit are rich in lipids. Differences in the fatty acid composition of seeds and fruits are clearly observed. Sea buckthorn seeds are rich in low‐chain (C4:0)–(C15:0) fatty acids, which were determined by the Soxhlet extraction method. The composition of the lipids obtained by US and SWE methods is practically identical and does not undergo transformation or an increase in the content of Elaidic acid methyl ester (C18:1n9t).

## Author Contributions

**Lana Datuashvili:** data curation (equal). **Maia Vanidze:** validation (equal). **Indira Japaridze:** investigation (equal). **Nona Surmanidze:** data curation (equal). **Inga Kartsivadze:** methodology (equal). **Ruslan Davitadze:** resources (equal). **Aleko Kalandia:** supervision (equal).

## Conflicts of Interest

The authors declare no conflicts of interest.

## Data Availability

Research data are not shared.
